# Dr. Harold Havelock Kynett

**Published:** 1917-12

**Authors:** 


**Dr. Harold Havelock Kynett,** U. of P., 1886, died in Philadelphia, Oct. 1, aged 55. For several years, he was editor in chief of the Medical and Surgical Reporter, the editor of the Buffalo Medical Journal then being on the Associate Staff of the Reporter. We pay due tribute to the manly qualities and professional and editorial ability of Dr. Kynett. Though not himself interested in that line of practice, the American Gastro-enterologic Assn. owes him a debt of gratitude for allowing the preliminary call for its organization meeting to be published in the Reporter and for offering the hospitality of its officers for the meeting, although for various reasons, this offer was not formally accepted. Unfortunately, our personal acquaintance with Dr. Kynett lapsed, through the unaccountable misunderstanding that he died several years ago.

